# Association between angiotensinogen (AGT), angiotensin-converting enzyme (ACE) and angiotensin-II receptor 1 (AGTR1) polymorphisms and COVID-19 infection in the southeast of Iran: a preliminary case-control study

**DOI:** 10.1186/s41231-021-00106-0

**Published:** 2021-11-17

**Authors:** Hamid Reza Kouhpayeh, Farhad Tabasi, Mohammad Dehvari, Mohammad Naderi, Gholamreza Bahari, Tahereh Khalili, Courtney Clark, Saeid Ghavami, Mohsen Taheri

**Affiliations:** 1 Infectious Diseases and Tropical Medicine Research Center, Resistant Tuberculosis Institute, Zahedan University of Medical Sciences, Zahedan, Iran; 2 Department of Physiology, Faculty of Medical Sciences, Tarbiat Modares University, Tehran, Iran; 3 Department of Clinical Biochemistry, School of Medicine, Zahedan University of Medical Sciences, Zahedan, Iran; 4 Genetics of Non-communicable Disease Research Center, Zahedan University of Medical Sciences, Zahedan, Iran; 5 Children and Adolescent Health Research Center, Resistant Tuberculosis Institute, Zahedan University of Medical Sciences, Zahedan, Iran; 6 Department of Human Anatomy and Cell Science, Max Rady College of Medicine, Rady Faculty of Health Sciences, University of Manitoba, Winnipeg, MB Canada; 7 Department of Genetic, School of Medicine, Zahedan University of Medical Sciences, Zahedan, 9816743463 Iran

**Keywords:** COVID-19, SARS-CoV-2, Angiotensin, AGT, ACE, AGTR1, Polymorphism

## Abstract

**Background:**

The COVID-19 pandemic remains an emerging public health crisis with serious adverse effects. The disease is caused by severe acute respiratory syndrome coronavirus-2 (SARS-CoV--2) infection, targeting angiotensin-converting enzyme-2 (ACE2) receptor for cell entry. However, changes in the renin-angiotensin system (RAS) balance alter an individual’s susceptibility to COVID-19 infection. We aimed to evaluate the association between AGT rs699 C > T, ACE rs4646994 I/D, and AGTR1 rs5186 C > A variants and the risk of COVID-19 infection and the severity in a sample of the southeast Iranian population.

**Methods:**

A total of 504 subjects, including 258 COVID-19 positives, and 246 healthy controls, were recruited. Genotyping of the ACE gene rs4646994, and AGT rs699, and AGTR1 rs5186 polymorphisms was performed by polymerase chain reaction (PCR) and PCR-restriction fragment length polymorphism (PCR-RFLP), respectively.

**Results:**

Our results showed that the II genotype of ACE rs4646994 and the I allele decreased the risk of COVID-19 infection. Moreover, we found that the TC genotype and C allele of AGT rs699 increased the risk of COVID-19 infection. The AGTR1 rs5186 was not associated with COVID-19 infection. Also, we did not find any association between these polymorphisms and the severity of the disease. However, we found a significantly higher age and prevalence of diabetes and hypertension in patients with severe disease than a non-severe disease.

**Conclusions:**

These findings suggest that ACE rs4646994 and AGT rs699 polymorphisms increase the risk of COVID-19 infection in a southeast Iranian population.

## Introduction

Coronavirus diseases-2019 (COVID-19) is an emerging global pandemic caused by severe acute respiratory syndrome coronavirus-2 (SARS-CoV-2). It was first identified in Wuhan, China, at the end of 2019 and has spread rapidly worldwide [1]. After about two years from the beginning of the pandemic, more than 235 million cases with approximately 4.8 million deaths are reported worldwide, with turned COVID-19 as a significant global crisis in modern history (https://www.worldometers.info/coronavirus). In Iran, beyond 5.5 million cases and 120,880 deaths due to COVID-19 are officially reported until the date. COVID-19 is mainly pneumonia characterized by cough, a fever, shortness of breath, fatigue, and gastrointestinal symptoms such as anorexia, nausea, vomiting, and diarrhea [2]; however, all organs could be affected by the virus, including the endocrine [3], cardiovascular [4], renal [5] and nervous systems [6] regardless the presence of pneumonia. SARS-CoV-2 is transmitted from human to human by many mechanisms such as respiratory droplets, aerosols, and unprotected contact [7].

SARS-CoV-2 is a highly infectious virus [8, 9] that uses angiotensin-converting enzyme 2 (ACE2) as the major receptor for viral entry in humans [10–13]. It has been shown that SARS-CoV-2 spike (S) glycoprotein binds via its receptor-binding domain (RDB) with a high affinity to human ACE2 [14], and therefore, mediates virus internalization [10, 15]. This mechanism is quite similar to the SARS-CoV virus [16], but SARS-CoV-2 has a remarkably higher affinity for ACE2 [17]. Therefore, recognizing features of the angiotensin pathway in COVID-19 can elucidate how individuals have differences in symptoms, severity, complications, and mortality. Understanding this mechanism could also be beneficial for therapeutic targeting. Potentially, an imbalance in this pathway, with the centrality of ACE1/ACE2 activity, can be responsible for COVID-19 pathophysiology [18–20].

Angiotensin II receptor 1 (AGTR1) may drive COVID-19 pathology: AGTR1 is G-protein coupled receptor (GPCR), mediates signaling and most functions of angiotensin-II that generated by the angiotensin-I converting enzyme (ACE1) [21, 22]. The angiotensin II via AGTR1 induces inflammation, apoptosis, and organ damage in pulmonary and cardiovascular tissues [23]. Previous evidence regarding SARS-CoV infection, mediated by ACE-2, which highly express in pulmonary tissue, indicates that binding SARS virus results in a decrease in ACE2 activity and expression [17], leading to an increase in angiotensin-II level [24]. Further, downregulated ACE2 decreases peptides converted from angiotensin-II to counteract its effects, including pro-apoptotic properties [18], inflammation, and fibrosis [19, 20], all of which are the basis of COVID-19 pathobiology.

On the other hand, genetic variations of a gene potentially alter the expression and functions of an encoded product, which can be considered the basis of inter-individual differences in susceptibility to infectious diseases [25]. Although the pandemic affected many populations, there are discrepancies regarding symptoms and disease severity, ranging from asymptomatic to devastating pneumonia with progressive multi-organ involvement. Part of these differences can be due to underlying conditions in at-risk populations; however, polymorphism in pathways related to the pathobiology of COVID-19, including angiotensin receptor and renin-angiotensin system (RAS), can be another contributing factor [26].

Angiotensinogen is a peptide hormone encoded by the *AGT* gene mapped on chromosome 1q42.2 [27]. The rs699 (M268T, previously known as M235T) is a missense polymorphism on exon 2, encodes the threonine variant, which is associated with increased angiotensin levels [28, 29]. Angiotensinogen is cleaved by renin, produce angiotensin-I, which is later converted to angiotensin-II by ACE1. ACE1 is encoded by the *ACE* gene on chromosome 17q23.3. The rs4646994 is a 287-bp insertion/deletion (indel) variant in intron 16 of the *ACE* gene, which is strongly correlated with ACE level [30] and activity [31]. Finally, angiotensin-II exerts its effects via AGTR1, encoded by the AGTR1 gene on chromosome 3q24. The rs5186 (A1166C) variant is located at the 3’untranlated region (3’UTR) of the *AGTR1* gene, potentially can affect mRNA stability and, therefore, AGTR1 levels [32], and associated with increased risk of hypertension [33].

Therefore, in addition to underlying known risk factors, host genetic predisposition may influence the risk, severity, and outcome of the disease [34]. Given the current evidence regarding the potential implication of angiotensin-related signaling cascade in COVID-19 [18, 19], we investigate the association between AGT rs699 C > T, ACE rs4646994 indel, and AGTR1 rs5186 C > A variants and the risk of COVID-19 infection in a sample of the southeast Iranian population.

## Materials and methods

### Subjects

The sample population for this case-control study comprised 258 COVID-19 subjects who tested positive for SARS-CoV-2 infection using real-time reverse transcription polymerase chain reaction (RT-PCR) technique and 246 healthy control subjects with similar geographic and ethnic backgrounds. According to World Health Organization (WHO) case definition, we enrolled patients who met with the definition for confirmed COVID-19 patient [35]. All patients were assessed thoroughly regarding preexisting conditions such as previous infectious diseases and underlying chronic conditions. All patients with a concurrent infectious disease were excluded. Samples were collected in Bu-Ali Sina Hospital, designated as a specialized center for infectious disease in Zahedan, from May 2020 through September 2020, in which the original SARS-CoV-2 was dominant. Further, the control group comprised individuals who tested negative to COVID-19 by RT-PCR method AND clinical diagnostic criteria, which finally ruled out the disease.

Severe and non-severe cases were defined based on WHO guidance for disease severity definition [35]. Accordingly, severe cases were defined as patients with a positive RT-PCR result for COVID-19, and clinical signs comply with severe pneumonia plus one of the following conditions: SpO2 < 90% on room air or respiratory rate > 30 breath/min or sign of severe respiratory distress. The non-severe case was defined as patients with a positive RT-PCR in the absence of any criteria for severe disease. Clinical diagnosis of participants was made by two infectious diseases specialists in Bu-Ali Sina Hospital. The study protocol was approved by the Ethics Committee of Zahedan University of Medical Sciences (IR.ZAUMS.REC.1399). Informed consent was provided by all subjects or their family members.

### Genotyping

**Fig. 1:**
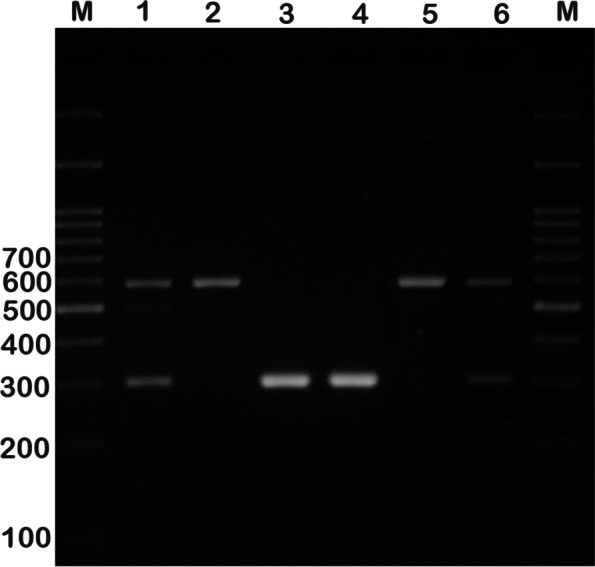
Electrophoresis pattern of the PCR products for ACE rs4646994 indel polymorphism detection. M: DNA marker; Lanes 1, 6: ID; Lanes 2, 5: II; Lanes 3, 4: DD

**Fig. 2:**
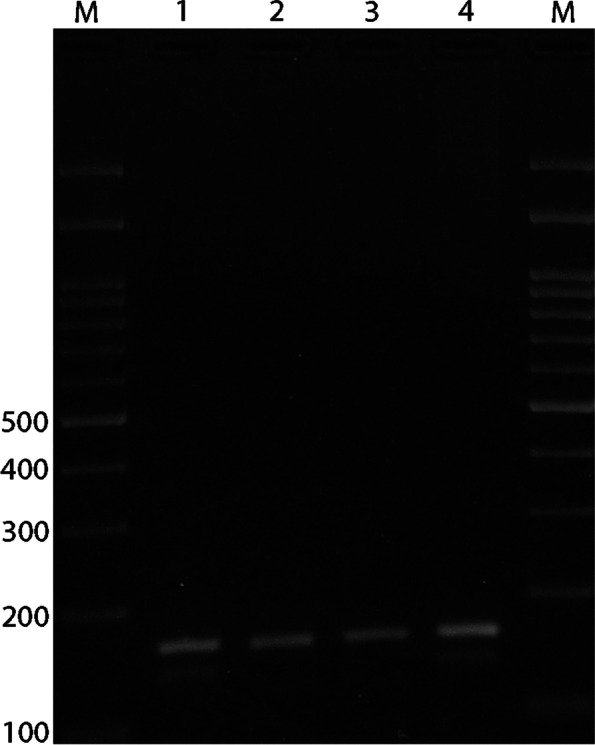
Electrophoresis pattern of the PCR-RFLP products for AGT rs699 polymorphism detection. M: DNA marker; Lanes 1, 4: TC; Lanes 2, 3: TT

**Fig. 3:**
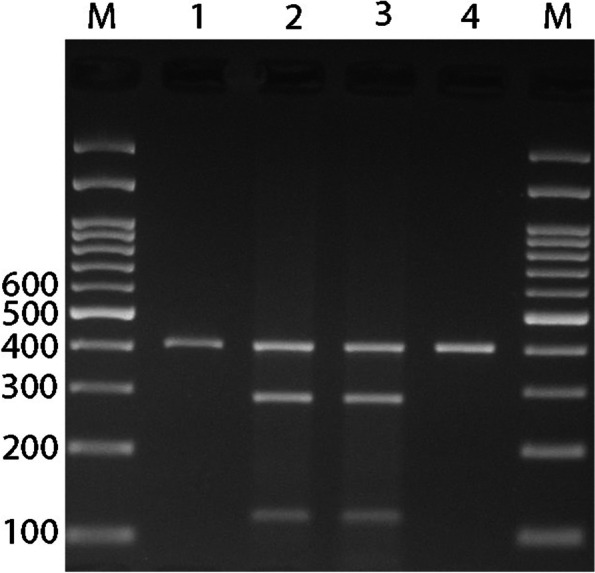
Electrophoresis pattern of the PCR-RFLP products for AGTR1 rs5186 polymorphism detection. M: DNA marker; Lanes 1, 4: AA; Lanes 2, 3: AC

Blood samples were drawn from each participant into a tube containing EDTA, and DNA was isolated using the salting-out method. We used PCR to detect ACE rs4646994 indel polymorphism. The PCR-restriction fragment length polymorphism (PCR-RFLP) method was applied for genotyping AGT rs699 and AGTR1 rs5186 polymorphisms. Primer sequences, restriction enzymes, and length of the fragments are summarized in Table 1. PCR was performed in a final volume of 20 μL containing 1 μL of genomic DNA (~ 100 ng/μL), 1 μL of each primer (10 μM), and 10 μL of 2X Prime Taq Premix (Genet Bio, Korea) and 7 μL ddH2O. PCR conditions included an initial denaturing step at 95 °C for 5 min, followed by 30 cycles of 95 °C for 30 s, annealing at 68 °C for AGT rs699, 60 °C for AT1R rs5186, and 66 °C for ACE rs4646994 for 30 s and 72 °C for 30 s, and a final extension at 72 °C for 5 min. The PCR product was digested by suitable restriction enzymes (Table 1). The fragments were then separated by electrophoresis in 2.5% agarose gels (Figs. 1, 2 and 3). We randomly selected 10% of the samples for quality control to repeat the results, which was 100% reproducible.

**Table 1:** Primer sequence used for detection of ACE rs4646994, AGT rs699 and AGTR1 rs5186 gene polymorphisms

Polymorphisms	Sequence (5`- > 3`)	Restriction Enzyme	Product size (bp)	Annealing temperature (°C)
**ACE rs4646994**	F: GCCCTGCAGGTGTCTGCAGCATGT R: GGATGGCTCTCCCCGCCTTGTCTC	–	I allele: 599 bp D allele: 312 bp	66
**AGT rs699**	F: CCGTTTGTGCAGGGCCTGGCTCTCT R: CAGGGTGCTGTCCACACTGGACCCC	Tth111I	T allele: 165 C allele: 141 + 24	68
**AGTR1 rs5186**	F: AGAAGCCTGCACCATGTTTTGAG R: CCTGTTGCTCCTCTAACGATTTA	DdeI	A allele: 410 bp C allele: 292 + 118	60

### Statistical analysis

Statistical analysis was performed using the SPSS 20.0 package software (IBM Corporation, USA). The Kolmogorov-Smirnov test was applied to assess the distribution normality. Comparisons were made by χ^2^ or independent sample *t*-test (or Mann-Whitney test, as appropriate) according to the data. Logistic regression analyses were used to calculate the odds ratios (ORs) and 95% confidence intervals (CIs) under different genetic models to explore the association between genotypes, COVID-19 infection risk, and disease severity. The level of statistical significance was defined as *p* < 0.05.

## Results

The study group included 258 confirmed COVID-19 patients (144 males, 114 females) and 246 healthy subjects (132 males, 114 females). The mean (SD) age of patients and controls were 50.23 (14.82) and 49.01 (14.95), respectively, and the median (IQR) of patients and control was 51.0 (23.0) and 48.5 (24.0), respectively. There was no statistically significant difference between the groups (*p* = 0.39). The demographic and clinical characteristics of patients are summarized in Table 2. Also, we compared COVID-19 patients based on the disease severity. Although there were no differences regarding sex between severe and non-severe cases (*p* = 0.31), patients with severe disease had significantly higher age than non-severe patients (*p* = 0.003). Additionally, we compared patients with established hypertension and diabetes (based on their medical records and investigations/examinations during hospitalization); the frequency of hypertension and diabetes was significantly higher in patients with severe disease (*p* < 0.001 and *p* = 0.042, respectively). The characteristics of severe and non-severe cases are summarized in Table 3.

**Table 2:** Demographic characteristics of COVID-19 patients and control individuals

Characteristics		Patients, n (%)	Control, n (%)	***p***
**Gender**	Male	144 (55.8)	132 (53.7)	0.34*
	Female	114 (44.2)	114 (46.3)	
	Total	258 (100)	246 (100)	
**Age**	Mean (SD)	50.23 (14.82)	49.01 (14.95)	0.39**
	Median (IQR)	51.0 (23.0)	48.5 (24.0)	

Analyzed by *χ^2^ and ** Mann-Whitney tests

Abbreviations: *SEM* standard error of mean, *IQR* interquartile range

**Table 3:** Demographic and clinical characteristics of severe and non-severe patients

Characteristics		Non-Severe, n(%)	Severe, n(%)	***p***
**Gender**	Male	55 (51.9)	89 (58.6)	0.31*
	Female	51 (48.1)	63 (41.4)	
	Total	106 (100)	152 (100)	
**Age**	Mean (SD)	45.69 (13.87)	52.27 (14.82)	0.003**
	Median (IQR)	46.0 (23.5)	52 (23.0)	
**Hypertension**	Yes	8 (16.7)	65 (43.9)	< 0.001*
	No	40 (83.3)	83 (56.1)	
	Total	48 (100)	148 (100)	
**Diabetes**	Yes	10 (20.8)	53 (35.3)	0.042*
	No	38 (79.2)	97 (64.7)	
	Total	48 (100)	150 (100)	

Analyzed by *χ^2^ and ** Mann-Whitney tests

Abbreviations: *SEM* standard error of mean, *IQR* interquartile range

The genotype and allele frequencies of ACE rs4646994, AGT rs699, and AGTR1 rs5186 are shown in Table 4. We calculated the Hardy-Winberg equilibrium (HWE) for patients and control subjects of each polymorphism. The results showed that distributions of ACE 4646994 and AGTR1 rs5186 (patients) were in HWE (*p* > 0.05), but distributions of AGTR1 rs5186 (controls) and AGT rs699 groups significantly deviated from HWE (*p* < 0.05, Table 4).

**Table 4:** Alleles and genotypes frequencies of ACE rs4646994, AGT rs699 and AGTR1 rs5186 polymorphisms in COVID-19 patients and control subjects

Polymorphism	Group	Genotypes, n (%)			Allele, n (%)		HWE	
							χ^**2**^	***p***
		**DD**	**DI**	**II**	**D**	**I**		
ACE rs4646994	Patients	144 (55.8)	89 (34.5)	25 (9.7)	377 (73.6)	139 (26.94)	3.94	0.052
	Controls	70 (28.7)	123 (50.4)	51 (20.9)	263 (53.89)	225 (46.11)	0.05	0.90
		**TT**	**TC**		**T**	**C**		
AGT rs699	Patients	20 (9.2)	197 (90.8)	–	237 (54.61)	197 (45.39)	142.9	< 0.001
	Controls	113 (46.1)	132 (53.9)	–	358 (73.06)	132 (26.94)	33.3	< 0.001
		**AA**	**AC**	**CC**	**A**	**C**		
AGTR1 rs5186	Patients	154 (74.4)	49 (23.7)	4 (1.9)	357 (86.81)	57 (13.19)	0.001	> 0.99
	Controls	185 (75.8)	59 (24.2)	0	429 (87.91)	59 (12.09)	4.61	0.031

Abbreviation: *HWE* Hardy-Weinberg equilibrium

### Association between the ACE rs4646994, AGT rs699, and AGTR1 rs5186 variants and risk of COVID-19 infection

The frequency of ACE rs4646994, AGT rs699, and AGTR1 rs5186 polymorphisms in COVID-19 patients and control subjects are shown in Table 4. The results revealed significant differences between the ACE rs4646994 and ATG rs699 polymorphisms, but not the ATGR1 rs5186 variant between patient and control groups. Our analyses of ACE rs4646994 shows that rs4646944 is associated with lower risk of COVID-19 in heterozygous (OR = 0.35, 95%CI = 0.23–0.52, *p* < 0.001, ID vs DD), homozygous (OR = 0.23, 95%CI = 0.13–0.41, *p* < 0.001, II vs DD), dominant (OR = 0.31, 95%CI = 0.21–0.46, *p* < 0.001, ID+DD vs DD), recessive (OR = 0.40, 95%CI = 0.24–0.67, *p* < 0.001, II vs ID+II), and overdominant (OR = 0.51, 95%CI = 0.36–0.74, *p* < 0.001, ID vs DD + II) models increased the risk of COVID-19 infection. In addition, the I allele was significantly associated with decreased risk of COVID-19 infection (OR = 0.43, 95%CI = 0.33–0.56, *p* < 0.001, I vs. D). These results showed that the D allele can be considered a risk factor for COVID-19 (Table 5). Genotyping of AGT rs699 revealed a remarkable difference between COVID-19 positive patients and healthy controls; TC genotype (OR = 8.43, 95% CI = 4.99–14.24, *p* < 0.001, TC vs. TT) as well as C allele (OR = 2.25, 95% CI = 1.71–2.96, *p* < 0.001, C vs. T) increased the risk of COVID-19 infection. As presented in Table 5, there was no significant difference in genotype and allelic distribution of AGTR1 rs5186 gene polymorphisms between COVID-19 patients and controls (*p* > 0.05). We performed the logistic regression adjusted for age and sex, which the result was the same as crude analysis (Table 5).

**Table 5:** Genotypes and allele frequencies of ACE rs4646994, ATG rs699 and AGTR1 rs5186 polymorphisms in COVID-19 patients and controls

Polymorphisms	Genetic Models	Patients, n (%)	Control, n (%)	Crude analysis		Adjusted analysis^**a**^	
**ACE rs4646994**	***Codominant***			**OR (95% CI)**	***p***	**OR (95% CI)**	***p***
	**DD**	144 (55.8)	70 (28.7)	1.00	–	1.00	–
	**ID**	89 (34.5)	123 (50.4)	**0.35 (0.23–0.52)**	**< 0.001**	**0.38 (0.24–0.60)**	**< 0.001**
	**II**	25 (9.7)	51 (20.9)	**0.23 (0.13–0.41)**	**< 0.001**	**0.11 (0.05–0.21)**	**< 0.001**
	***Dominant***						
	**DD**	144 (55.8)	70 (28.7)	1.00	–	1.00	–
	**ID + II**	114 (44.2)	174 (71.3)	**0.31 (0.21–0.46)**	**< 0.001**	**0.27 (0.18–0.42)**	**< 0.001**
	***Recessive***						
	**DD + ID**	233 (90.3)	193 (79.1)	1.00	–	1.00	–
	**II**	25 (9.7)	51 (20.9)	**0.40 (0.24–0.67)**	**< 0.001**	**0.18 (0.09–0.34)**	**< 0.001**
	**Overdominant**						
	**DD + II**	169 (65.5)	121 (49.6)	1.00	–	1.00	–
	**ID**	89 (34.5)	123 (50.4)	**0.51 (0.36–0.74)**	**< 0.001**	0.71 (0.47–1.06)	0.058
	***Alleles***						
	**D**	377 (73.6)	263 (53.89)	1.00	–	1.00	–
	**I**	139 (26.94)	225 (46.11)	**0.43 (0.33–0.56)**	**< 0.001**	**0.32 (0.24–0.43)**	**< 0.001**
**ATG rs699**	***Codominant***						
	**TT**	20 (9.2)	113 (46.1)	1.00	–		
	**TC**	197 (90.8)	132 (53.9)	**8.43 (4.99–14.24)**	**< 0.001**	**18.17 (9.88–33.39)**	**< 0.001**
	***Alleles***						
	**T**	237 (54.61)	358 (73.06)	1.00	–	1.00	–
	**C**	197 (45.39)	132 (26.94)	**2. 25 (1.71–2.96)**	**< 0.001**	**3.61 (2.58–5.05)**	**< 0.001**
**ATGR1 rs5186**	***Codominant***						
	**AA**	154 (74.4)	185 (75.8)	1.00	–	1.00	–
	**AC**	49 (23.7)	59 (24.2)	0.99 (0.64–1.54)	0.5	0.83 (0.51–1.36)	0.28
	**CC**	4 (1.9)	0	–	–	–	–
	**Dominant**						
	**AA**	154 (74.4)	185 (75.8)	1.00	–	1.00	–
	**AC + CC**	53 (25.6)	59 (24.2)	1.07 (0.70–1.65)	0.40	0.90 (0.55–1.45)	0.38
	**Recessive**						
	**AA + AC**	203 (98.1)	244 (100)	1.00	–	1.00	–
	**CC**	4 (1.9)	0	–	–	–	–
	**Overdominant**						
	**AA + CC**	158 (76.3)	185 (75.8)	1.00	–	1.00	–
	**AC**	49 (23.7)	59 (24.2)	0.97 (0.62–1.50)	0.49	0.82 (0.50–1.33)	0.24
	***Alleles***						
	**A**	357 (86.81)	429 (87.91)	1.00	–	1.00	–
	**C**	57 (13.19)	59 (12.09)	1.10 (0.74–1.63)	0.34	0.99 (0.64–1.52)	0.52

^a^Adjusted for age and sex

### Association between the ACE rs4646994, AGT rs699, and AGTR1 rs5186 variants and the severity of COVID-19 infection

We compared the prevalence of ACE rs4646994, AGT rs699, and AGTR1 rs5186 polymorphisms between severe and non-severe cases of COVID-19 (Table 6). As presented in Table 6, there was no significant difference in genotype and allelic distribution of ACE rs4646994, AGT rs699, and AGTR1 rs5186 gene polymorphisms and severity of the disease. However, ACE rs4646994 showed a significant association with non-severe disease in recessive model (OR = 0.42, 95%CI = 0.18–0.99, *p* = 0.03, II vs DD + ID). Also, the C allele of AGTR1 rs5186 was a significant association with non-severe disease (OR = 0.51, 95%CI = 0.29–0.90, *p* = 0.01, C vs. A). These trends were not observed in other hereditary models (Table 6). Further, we performed logistic regression adjusted for age, sex, and existence for diabetes and hypertension. The results were the same as the crude analyses regarding the association between genetic models and severity (Table 6).

**Table 6:** Genotypes and allele frequencies of ACE rs4646994, ATG rs699 and AGTR1 rs5186 polymorphisms in non-severe and severe COVID-19 patients

Polymorphisms	Genetic Models	Severe, n (%)	Non-severe, n (%)	Crude analysis		Adjusted analysis^**a**^	
				OR (95% CI)	***p***	OR (95% CI)	***p***
**ACE rs4646994**	***Codominant***						
	**DD**	84 (55.3)	60 (56.6)	1.00	–	1.00	–
	**ID**	58 (38.2)	31 (29.2)	1.33 (0.77–2.31)	0.18	1.12 (0.57–2.21)	0.42
	**II**	10 (6.6)	15 (14.2)	0.47 (0.20–1.13)	0.06	0.70 (0.14–3.45)	0.49
	***Dominant***						
	**DD**	84 (55.3)	60 (56.6)	1.00	–	1.00	–
	**ID + II**	68 (44.7)	46 (43.4)	1.05 (0.64–1.74)	0.46	1.06 (0.55–2.05)	0.48
	***Recessive***						
	**DD + ID**	142 (93.4)	91 (85.8)	1.00	–	1.00	–
	**II**	10 (6.6)	15 (14.2)	**0.42 (0.18–0.99)**	**0.03**	0.66 (0.13–3.19)	0.46
	**Overdominant**						
	**DD + II**	94 (61.8)	75 (70.8)	1.00	–	1.00	–
	**ID**	58 (38.2)	31 (29.2)	1.49 (0.87–2.53)	0.08	1.16 (0.59–2.25)	0.39
	***Alleles***						
	**D**	226 (74.5)	151 (75.0)	1.00	–	1.00	–
	**I**	78 (25.50)	61 (25.0)	0.85 (0.57–1.26)	0.24	0.99 (0.58–1.68)	0.54
**ATG rs699**	***Codominant***						
	**TT**	8 (6.3)	12 (13.3)	1.00	–	1.00	–
	**TC**	119 (93.7)	78 (86.7)	2.28 (0.89–5.85)	0.06	2.33 (0.75–7.14)	0.11
	***Alleles***						
	**T**	135 (53.15)	102 (56.67)	1.00	–	1.00	–
	**C**	119 (46.85)	78 (43.44)	1.15 (0.78–1.69)	0.26	1.16 (0.70–1.90)	0.31
**ATGR1 rs5186**	***Codominant***						
	**AA**	96 (80.0)	58 (66.7)	1.00	–	1.00	–
	**AC**	23 (19.2)	26 (29.9)	0.53 (0.27–1.02)	0.06	0.55 (0.24–1.25)	0.11
	**CC**	1 (0.8%)	3 (3.5)	0.20 (0.02–1.98)	0.16	0.30 (0.01–4.97)	0.41
	**Dominant**						
	**AA**	95 (80	58 (66.7)	1.00	–	1.00	–
	**AC + CC**	24 (20.0)	19 (35.3)	0.77 (0.38–1.52)	0.28	0.53 (0.23–1.18)	0.09
	**Recessive**						
	**AA + AC**	119 (99.2)	84 (96.5)	1.00	–	1.00	–
	**CC**	1 (0.8)	3 (3.5)	23 (0.02–2.30)	0.20	0.34 (0.02–5.69)	0.45
	**Overdominant**						
	**AA + CC**	97 (80.8)	61 (70.1)	1.00	–	1.00	–
	**AC**	23 (19.2)	26 (29.2)	0.55 (0.29–1.06)	0.052	0.56 (0.24–1.28)	0.12
	***Alleles***						
	**A**	215 (89.58)	142 (81.61)	1.00	–		
	**C**	25 (10.42)	32 (18.39)	**0.51 (0.29–0.90)**	**0.01**	0.56 (0.27–1.14)	0.08

^a^Adjusted for age, sex, hypertension and diabetes

## Discussion

In the present study, we explored the association of three important variants in the angiotensin pathway with susceptibility to COVID-19 in patients from southeast Iran. Our result showed that ACE indel and ATG rs699 is associated with susceptibility to COVID-19 infection; however, we did not observe any association between these polymorphisms and the risk of severe disease, but the age and prevalence of diabetes and hypertension in patients with severe disease were remarkably higher than patients with a non-severe disease.

COVID-19 infection has a variety of symptoms and severity in different individuals. Symptoms of patients infected with SARS-CoV-2 range from entirely asymptomatic to mild constitutional symptoms, which are commonly manifesting with fever, cough, and fatigue, to severe pneumonia associated with acute respiratory distress syndrome (ARDS), systemic inflammation, and organ failure [36, 37]. The latter frequently necessitated hospitalization and intensive care [38]. Certain individuals are at increased risk for developing more symptomatic and severe illnesses. Previous studies introduced older ages [39] and specific medical conditions, including diabetes and hypertension, as major risk factors for more severe disease [38, 40, 41]. This was consistent with our results that demonstrated a higher prevalence of hypertension and diabetes in severe COVID-19, as well as older age.

On the other hand, other host factors, such as genetic variations, can be responsible for the observed differences in susceptibility and disease severity. Mounting evidence indicates that the RAS imbalance, which is closely associated with ARDS [42], plays a key role in COVID-19 pathophysiology [43]. SARS-CoV-2 binds to ACE-II surface receptors, one of the major components of RAS for cell entrance in humans [10, 11]. Angiotensinogen converted by renin to angiotensin-I and then by ACE to angiotensin-II. Notably, angiotensin-II, via binding to AGTR1, induces a pro-inflammatory state, vasoconstriction, and subsequently fibrosis. ACE2, the known target surface receptor of SARS-CoV-2, converts angiotensin-II to angiotensin 1–7. Angiotensin 1–7 has contradictory effects to angiotensin-II, such as anti-inflammatory and vasodilatory actions [44]. Binding SARS-CoV-2 to ACE2 can change the ACE/ACE2 balance, which may increase angiotensin-II levels, and further increase its harmful effects, mainly in lung tissue [45, 46]. This process can be the starting point of ARDS and subsequent cytokine overproduction and over-activation, known as the cytokine storm. Patients with underlying diabetes and hypertension show a decreased ACE2 expression state, associated with increased angiotensin-II and a pro-inflammatory condition [47]. Therefore, a more severe COVID-19 may occur in these patients [48].

Any alteration in expression and function of RAS elements, for instance, caused by genetic variations, can results in differences in susceptibility to COVID-19 pathogenesis. Previously reported that the C allele (threonine variant) of AGT rs699 is associated with increased plasma angiotensinogen and hypertension [29, 49, 50]. Therefore, this variant potentially could be associated with increased susceptibility to COVID-19. Consistent with this hypothesis, we observed that TC genotype and C allele of rs699 were associated with 8.4- and 2.2-fold increase in the risk of COVID-19 compared to TT genotype and T allele, respectively. Although patients with severe disease in our study had a higher prevalence of TC genotype and C allele, we did not observe a statistically significant difference, which could be due to the relatively small sample size.

The ACE rs4646994 is a common indel polymorphism with 287-bp *Alu*-type sequence at the intron 16 position, leading to higher ACE activity and serum levels and angiotensin-II levels in D carriers [51, 52]. This elevated level could be a strong risk factor for cardiovascular and renal diseases [53]. Also, a positive correlation between the D allele, ACE levels, and ARDS has been observed [54]. However, its association with hypertension seems to be ethnic-dependent [55]. Carriers of the D allele are reported to have a higher risk for COVID-19 infection, severity, and outcome [26, 56]. In a study on the Asian population, Pati et al. revealed a positive correlation between the D allele and SARS-CoV-2 infection [57]. Verma et al. reported that the DD genotype, D allele, diabetes, and hypertension were significantly higher in severe cases of COVID-19 [58]. Yamamoto et al. also showed that the II genotype has a strong negative correlation with COVID-19 prevalence and death [59]. Similar to previous findings, we observed a marked decrease of COVID-19 risk in carriers of the I allele in the present study. The presence of the I allele significantly reduced the risk of COVID-19 in all hereditary models, which means that the D allele is a risk factor for COVID-19 infection. Further, we found that II genotype in recessive model, decreases the risk of severe disease. Although not statistically significant, the same trend was observed in codominant model.

The ATGR1 rs5186 variant is one of the most extensively studied polymorphisms associated with essential hypertension [33]. This polymorphism alters the transcription of impairs the binding of target microRNA (i.e., miR-155), leading to increased expression of the *AGTR1* gene [60]. Thus, it could be associated with a higher response to angiotensin-II. However, we could not find any significant association between rs5186 and susceptibility to COVID-19 in our studied sample population.

In conclusion, our results suggest ACE rs4646994 and AGT rs699 are associated with the risk of COVID-19 infection and may be used as predictive biomarkers, for instabce the diagnosis of patients at higher risk for developing COVID-19 infection. We found that ACE rs4646994 I allele and AGT C allele are associated with decreased and increased risk of COVID-19 infection, respectively. In addition, age, diabetes, and hypertension are risk factors for severe COVID-19 infection. Further studies with a larger sample size in different populations are required to validate our findings.

## Data Availability

All data of the manuscript will be provided upon reasonable request and approval by the ethics committee.

## References

[1] Bellone M, Calvisi SL (2020). ACE polymorphisms and COVID-19-related mortality in Europe. J Mol Med (Berl).

[2] Zhang H (2021). Clinical characteristics of coronavirus disease 2019 (COVID-19) in patients out of Wuhan from China: a case control study. BMC Infect Dis.

[3] Lundholm MD (2020). SARS-CoV-2 (COVID-19) and the Endocrine System. J Endocr Soc.

[4] Azevedo RB (2021). Covid-19 and the cardiovascular system: a comprehensive review. J Hum Hypertens.

[5] Kaye AD (2021). COVID-19 impact on the renal system: pathophysiology and clinical outcomes. Best Pract Res Clin Anaesthesiol.

[6] Iadecola C, Anrather J, Kamel H (2020). Effects of COVID-19 on the Nervous System. Cell.

[7] Jayaweera M (2020). Transmission of COVID-19 virus by droplets and aerosols: a critical review on the unresolved dichotomy. Environ Res.

[8] Sanche S (2020). High contagiousness and rapid spread of severe acute respiratory syndrome coronavirus 2. Emerg Infect Dis.

[9] Peymani P (2021). Statins in patients with COVID-19: a retrospective cohort study in Iranian COVID-19 patients. Transl Med Commun.

[10] Parit R, Jayavel S (2021). Association of ACE inhibitors and angiotensin type II blockers with ACE2 overexpression in COVID-19 comorbidities: a pathway-based analytical study. Eur J Pharmacol.

[11] Hoffmann M (2020). SARS-CoV-2 Cell Entry Depends on ACE2 and TMPRSS2 and Is Blocked by a Clinically Proven Protease Inhibitor. Cell.

[12] Siri M (2021). Autophagy, Unfolded Protein Response, and Neuropilin-1 Cross-Talk in SARS-CoV-2 Infection: What Can Be Learned from Other Coronaviruses. Int J Mol Sci.

[13] Peng R (2021). Cell entry by SARS-CoV-2. Trends Biochem Sci.

[14] Lu J, Sun PD (2020). High affinity binding of SARS-CoV-2 spike protein enhances ACE2 carboxypeptidase activity. J Biol Chem.

[15] Sharma P (2021). Chloroquine: autophagy inhibitor, antimalarial, bitter taste receptor agonist in fight against COVID-19, a reality check?. Eur J Pharmacol.

[16] Li W (2003). Angiotensin-converting enzyme 2 is a functional receptor for the SARS coronavirus. Nature.

[17] Shang J (2020). Structural basis of receptor recognition by SARS-CoV-2. Nature.

[18] Sriram K, Insel PA (2020). A hypothesis for pathobiology and treatment of COVID-19: the centrality of ACE1/ACE2 imbalance. Br J Pharmacol.

[19] Sriram K, Loomba R, Insel PA (2020). Targeting the renin− angiotensin signaling pathway in COVID-19: unanswered questions, opportunities, and challenges. Proc Natl Acad Sci.

[20] Drozdzal S (2020). FDA approved drugs with pharmacotherapeutic potential for SARS-CoV-2 (COVID-19) therapy. Drug Resist Updat.

[21] Nabavi SF (2021). Rationale for effective prophylaxis against COVID-19 through simultaneous blockade of both Endosomal and non-Endosomal SARS-CoV-2 entry into host cell. Clin Transl Sci.

[22] Darbeheshti F (2021). Coronavirus: pure infectious disease or genetic predisposition. Adv Exp Med Biol.

[23] Forrester SJ (2018). Angiotensin II signal transduction: an update on mechanisms of physiology and pathophysiology. Physiol Rev.

[24] Haga S (2008). Modulation of TNF-α-converting enzyme by the spike protein of SARS-CoV and ACE2 induces TNF-α production and facilitates viral entry. Proc Natl Acad Sci.

[25] Burgner D, Jamieson SE, Blackwell JM (2006). Genetic susceptibility to infectious diseases: big is beautiful, but will bigger be even better?. Lancet Infect Dis.

[26] Delanghe JR, Speeckaert MM, De Buyzere ML (2020). The host's angiotensin-converting enzyme polymorphism may explain epidemiological findings in COVID-19 infections. Clin Chim Acta.

[27] Corvol P, Jeunemaitre X (1997). Molecular genetics of human hypertension: role of angiotensinogen*. Endocr Rev.

[28] Brand E (2002). Detection of putative functional angiotensinogen (AGT) gene variants controlling plasma AGT levels by combined segregation-linkage analysis. Eur J Hum Genet.

[29] Sethi AA, Nordestgaard BG, Tybjærg-Hansen A (2003). Angiotensinogen gene polymorphism, plasma angiotensinogen, and risk of hypertension and ischemic heart disease: a meta-analysis. Arterioscler Thromb Vasc Biol.

[30] Rigat B (1990). An insertion/deletion polymorphism in the angiotensin I-converting enzyme gene accounting for half the variance of serum enzyme levels. J Clin Invest.

[31] Rigat B (1992). PCR detection of the insertion/deletion polymorphism of the human angiotensin converting enzyme gene (DCP1)(dipeptidyl carboxypeptidase 1). Nucleic Acids Res.

[32] Abdollahi MR (2007). Quantitated transcript haplotypes (QTH) of AGTR1, reduced abundance of mRNA haplotypes containing 1166C (rs5186: a> C), and relevance to metabolic syndrome traits. Hum Mutat.

[33] Mottl AK, Shoham DA, North KE (2008). Angiotensin II type 1 receptor polymorphisms and susceptibility to hypertension: a HuGE review. Genet Med.

[34] Hashemi SMA, et al. Human gene polymorphisms and their possible impact on the clinical outcome of SARS-CoV-2 infection. Arch Virol. 2021;166(8):2089–108. 10.1007/s00705-021-05070-6 PMC8088757 33934196

[35] Organization, W.H. COVID-19 clinical management: living guidance, 25 January 2021. Geneva: World Health Organization; 2021.

[36] Hu Y (2020). Prevalence and severity of corona virus disease 2019 (COVID-19): a systematic review and meta-analysis. J Clin Virol.

[37] Singhavi H, et al. SARS-Cov2: a meta-analysis of symptom distribution by continent in 7310 adult COVID-19 infected patients. Virusdisease. 2021;32(3):400–9. 10.1007/s13337-021-00699-y PMC8187893 34124318

[38] Vahedi A (2020). Clinical features and outcomes of ICU patients with COVID-19 infection in Tehran, Iran: a single-centered retrospective cohort study. Tanaffos.

[39] Incerti D (2021). Prognostic model to identify and quantify risk factors for mortality among hospitalised patients with COVID-19 in the USA. BMJ Open.

[40] McGurnaghan SJ (2021). Risks of and risk factors for COVID-19 disease in people with diabetes: a cohort study of the total population of Scotland. Lancet Diabetes Endocrinol.

[41] Gao YD (2021). Risk factors for severe and critically ill COVID-19 patients: a review. Allergy.

[42] Vrigkou E (2017). The evolving role of the renin-angiotensin system in ARDS. Crit Care.

[43] Henry BM (2020). Hyperinflammation and derangement of renin-angiotensin-aldosterone system in COVID-19: A novel hypothesis for clinically suspected hypercoagulopathy and microvascular immunothrombosis. Clin Chim Acta.

[44] Bader M (2013). ACE2, angiotensin-(1–7), and mas: the other side of the coin. Pflügers Arch.

[45] Tikellis C, Thomas M. Angiotensin-converting enzyme 2 (ACE2) is a key modulator of the renin angiotensin system in health and disease. Int J Pept. 2012;2012:1–8. 10.1155/2012/256294 PMC3321295 22536270

[46] Liu Y (2020). Clinical and biochemical indexes from 2019-nCoV infected patients linked to viral loads and lung injury. Sci China Life Sci.

[47] Di Raimondo D (2012). Effects of ACE-inhibitors and angiotensin receptor blockers on inflammation. Curr Pharm Des.

[48] Guan W-J (2020). Clinical characteristics of coronavirus disease 2019 in China. N Engl J Med.

[49] Takeuchi F (2012). Reevaluation of the association of seven candidate genes with blood pressure and hypertension: a replication study and meta-analysis with a larger sample size. Hypertens Res.

[50] Jeunemaitre X (1992). Molecular basis of human hypertension: role of angiotensinogen. Cell.

[51] Staessen JA (1997). The deletion/insertion polymorphism of the angiotensin converting enzyme gene and cardiovascular-renal risk. J Hypertens.

[52] Danser AJ (2007). ACE phenotyping as a first step toward personalized medicine for ACE inhibitors. Why does ACE genotyping not predict the therapeutic efficacy of ACE inhibition?. Pharmacol Ther.

[53] Rudnicki M, Mayer G (2009). Significance of genetic polymorphisms of the renin-angiotensin-aldosterone system in cardiovascular and renal disease. Pharmacogenomics.

[54] Marshall RP (2002). Angiotensin converting enzyme insertion/deletion polymorphism is associated with susceptibility and outcome in acute respiratory distress syndrome. Am J Respir Crit Care Med.

[55] Ned RM (2012). The ACE I/D polymorphism in US adults: limited evidence of association with hypertension-related traits and sex-specific effects by race/ethnicity. Am J Hypertens.

[56] Sarangarajan R (2021). Ethnic prevalence of angiotensin-converting enzyme deletion (D) polymorphism and COVID-19 risk: rationale for use of angiotensin-converting enzyme inhibitors/angiotensin receptor blockers. J Racial Ethn Health Disparities.

[57] Pati A (2020). ACE deletion allele is associated with susceptibility to SARS-CoV-2 infection and mortality rate: an epidemiological study in the Asian population. Clin Chim Acta.

[58] Verma S (2021). Impact of I/D polymorphism of angiotensin-converting enzyme 1 (ACE1) gene on the severity of COVID-19 patients. Infect Genet Evol.

[59] Yamamoto N (2020). SARS-CoV-2 infections and COVID-19 mortalities strongly correlate with ACE1 I/D genotype. Gene.

[60] Sethupathy P (2007). Human microRNA-155 on chromosome 21 differentially interacts with its polymorphic target in the AGTR1 3′ untranslated region: a mechanism for functional single-nucleotide polymorphisms related to phenotypes. Am J Hum Genet.

